# The impact of esophageal device insertion on cuff pressure of endotracheal tube: a literature review and meta-analysis

**DOI:** 10.1038/s41598-022-21980-0

**Published:** 2022-10-28

**Authors:** Kuo-Chuan Hung, Ying-Jen Chang, Yang-Pei Chang, Chun-Ning Ho, Kuo-Mao Lan, Jen-Yin Chen, Li-Kai Wang, Ping-Wen Huang, Cheuk-Kwan Sun

**Affiliations:** 1 Department of Anesthesiology, Chi Mei Medical Center, Tainan City, Taiwan; 2 Department of Hospital and Health Care Administration, College of Recreation and Health Management, Chia Nan University of Pharmacy and Science, Tainan City, Taiwan; 3 Department of Recreation and Health-Care Management, College of Recreation and Health Management, Chia Nan University of Pharmacy and Science, Tainan City, Taiwan; 4 Department of Neurology, Kaohsiung Municipal Ta-Tung Hospital, Kaohsiung Medical University, Kaohsiung City, Taiwan; 5 Department of Neurology, Kaohsiung Medical University Hospital, Kaohsiung Medical University, Kaohsiung City, Taiwan; 6 Department of Emergency Medicine, Show Chwan Memorial Hospital, Changhua City, Taiwan; 7 Department of Emergency Medicine, E-Da Hospital, No.1, Yida Road, Jiaosu Village, Yanchao District, Kaohsiung City, 82445 Taiwan; 8 College of Medicine, I-Shou University, Kaohsiung City, Taiwan

**Keywords:** Medical research, Risk factors

## Abstract

The impact of intraoperative esophageal device insertion (EDI) on endotracheal tube (ET) cuff inflation pressure remains unclear. Electronic databases including Medline, Embase, Google scholar, Web of Science™ and Cochrane Central Register of Controlled Trials were searched for studies involving EDI after placement of ETs from inception to July 7, 2022. The primary outcome was risk of high cuff pressure, while the secondary outcomes were increases in cuff pressure following EDI. Difference between adults and children was investigated with subgroup analysis. There were ten eligible studies (observation study, n = 9, randomized controlled study, n = 1) involving a total of 468 participants. EDI notably increased the risk of high cuff pressure (n = 7, risk ratio: 12.82, 95% confidence interval: 4.9 to 33.52, subgroup analysis: *p* = 0.008). There were significant elevations in cuff pressure in adults and children both during (13.42 and 7.88 cmH_2_O, respectively, subgroup analysis: *p* = 0.15) and after (10.09 and 3.99 cmH_2_O, respectively, subgroup analysis: *p* = 0.0003) EDI. Our results revealed an over 12-fold increase in the risk of high endotracheal tube cuff pressure in patients, especially adults, receiving EDI under endotracheal anesthesia. There were significant increases in both adults and children despite a higher increase in the former after device insertion.

## Introduction

Maintaining the cuff pressure of endotracheal tube (ETT) within a suitable range is of paramount importance as under- or over-inflation could be associated with clinically significant complications^1^; while under-inflation may result in ineffective sealing of the tracheal opening and an elevated risk of pulmonary aspiration, over-inflation may compromise tracheal mucosal circulation and result in tracheal injury^2–5^. Over-inflation of an ETT cuff is defined as the injection of a volume of air more than that needed to create an adequate seal between the cuff and the tracheal wall^6^. It is well known that tracheal injury is correlated with cuff pressure as compromise of tracheal mucosal blood flow is an important contributor to intubation-related tracheal morbidity^2^.

Previous studies have shown that a cuff pressure of > 30 cmH_2_O may impede local tissue blood flow and cause damage to the tracheal mucosal wall as well as the surrounding anatomical structures^6,7^, resulting in complications ranging from sore throat^8,9^, hoarseness, recurrent laryngeal nerve injuries^10^, tracheal ulceration, necrosis, stenosis^11^, the formation of tracheal diverticulum^12^ and tracheo-esophageal fistula^13–18^ to the life-threatening condition of tracheal rupture during cardiac resuscitation^19^. Besides, evidence from animal experiments demonstrated consistent tracheal mucosal damage even only after a brief exposure to an over-inflated tracheal cuff^20^. Physiologically, blood flow to the antero-lateral part of the trachea has been reported to be compromised at pressures exceeding 30 cmH_2_O and become obstructed at pressures exceeding 50 cmH_2_O^21^. Previous studies have also shown that hyperinflation of ETT cuff could result in the herniation of the cuff balloon in front of the tube’s end^22^ or upwards through the glottis^23^, thereby jeopardizing gas exchange. Hence, general practice guidelines recommended a cuff inflation pressure below 30 cmH_2_O (22 mmHg)^7^.

Despite careful monitoring of cuff pressure after tracheal intubation for anesthesia, intra-operative insertion of esophageal devices may alter the cuff pressure^24^. Indeed, previous studies have revealed that insertion of a medical device into the esophagus, which is situated between the rigid cervical spine and the trachea, in a patient under endotracheal anesthesia may increase ETT cuff pressure^24–31^. In addition, the use of a bougie in patients undergoing bariatric surgery may be related to an increased risk of esophageal complications^32,33^, while the insertion of a transesophageal echocardiography (TEE) probe during cardiac surgery may be associated with additional risks of airway complications (e.g., ETT obstruction)^34,35^. However, the clinical significance of such impacts in adults and children has not been systematically reviewed based on pooled evidence.

Therefore, the present meta-analysis aimed at elucidating the risk of high cuff pressure in patients undergoing endotracheal general anesthesia for procedures involving the insertion of esophageal devices. We also investigated the increases in cuff pressure during and after their insertion and compared the differences between adults and children in an attempt to provide evidence-based guidance for clinical practice.

## Materials and methods

### Guidelines and registration

This meta-analysis was conducted based on the recommendation of the Preferred Reporting Items for Systematic Reviews and Meta-Analyses (PRISMA) statement and was registered with PROSPERO (CRD42021232644).

### Search strategy

The databases of Medline, Embase, Google scholar, Web of Science™ and the Cochrane Central Register of Controlled Trials (CENTRAL) were searched for reports using the keywords: ("Tracheal intubation" or "Endotracheal intubation" or "general anesthesia") AND ("Nasogastric tube*" or "NG tube*" or "Bougie*" or "Transesophageal echocardiography probe*" or "Orogastric tube*" or "Probe*" or "Transesophageal probe*" or "esophagogastroduodenoscopy probe*" or "Esophageal Stethoscope*" or "Gastrointestinal Intubation" or "Nasogastric Intubations") AND ("Cuff pressure*" or "Intracuff pressure*" or "intracuff measurement") from inception to April 11, 2021 (Updated on July 7, 2022). Subject headings (e.g., MeSH terms in Cochrane Library) were also used to assist in searching. We manually searched the Google scholar and references included in all the retrieved articles to identify potentially eligible studies not identified during our electronic screening. No restriction on publication date was applied, but only studies published in English were reviewed.

### Inclusion and exclusion criteria

The abstracts and titles of the retrieved studies were independently screened by two investigators who also read the full text of the potentially eligible articles and discussed the contents. Conflicts were resolved by a third investigator. Another investigator screened additional references from the included articles. The criteria for eligibility of studies included: (1) patients receiving tracheal intubation with cuffed ETTs, (2) studies involving esophageal insertion of medical devices after the ETT placement, (3) available data regarding change in cuff pressure. The exclusion criteria were (1) studies with unavailable information about changes in cuff pressure, (2) the use of nitrous oxide for maintenance of general anesthesia as nitrous oxide is associated with an increase in endotracheal cuff pressure^2^, and (3) articles not formally published (e.g., those in Research Square). Two authors independently investigated the eligibility of the selected trials for final analysis, while two other reviewers independently extracted necessary data. On encountering disagreements, a third author was consulted to reach a consensus. We contacted the corresponding authors of trials that did not provide data on primary or secondary outcomes to retrieve the missing information.

### Primary outcome and secondary outcomes

The primary outcome was the risk of high cuff pressure, the definition of which was according to that of each study. The secondary outcomes were the increases in cuff pressure during and after EDI. Subgroup analyses were also performed to investigate the difference between adults and children as well as that between cardiac and non-cardiac surgery in adult patients.

### Risk of bias assessment

Studies deemed eligible were assessed for methodological quality and risk of bias by two independent reviewers using the Cochrane Collaboration risk of bias tool^36^, and the Newcastle–Ottawa Scale (NOS)^37^ for randomized controlled trials and comparative studies (cohorts and case–control studies), respectively. The NOS for observational studies was based on three domains, namely, study group selection, group comparability, and outcome of interest ascertainment. For the Selection, Comparability, and Outcome domains, a maximum of four, two, and three stars could be assigned, respectively. A higher number of stars denotes a better quality of the study with nine stars indicating the highest quality^37^. A study with a low risk of bias was defined as one having seven stars or more. Disagreements were resolved through discussion.

### Statistical analysis

Based on the random effects model, the risk ratios (RRs) with 95% confidence intervals (CIs) were computed for dichotomous outcomes which were pooled with the Mantel–Haenszel (MH) method^38,39^. On the other hand, the mean difference (MD) represented the effect size for continuous outcomes. The I^2^ statistic was used to assess the degree of variability in effect estimates attributable to heterogeneity rather than error in sampling. We also conducted sensitivity analyses to test the robustness of our findings by omitting one trial at a time from the meta-analysis to evaluate the potential influence of a particular study on the overall outcomes. A funnel plot was examined for symmetry to assess the probabilities of publication and reporting bias on encountering 10 or more studies reporting on a specific outcome. To assess the impact of demographic characteristics on changes in cuff pressure in adult patients, univariate analysis with a meta-regression approach was performed through including one covariate at a time [i.e., age, prevalence of male gender, body mass index (BMI)]. For all analyses, we set the significance level at 0.05 and used the Cochrane Review Manager (RevMan 5.4; Copenhagen: The Nordic Cochrane Center, The Cochrane Collaboration, 2014) for data synthesis. Meta-regression was conducted using the Open Meta-Analyst software (Brown University, Providence, RI; http://www.cebm.brown.edu/openmeta/).

## Results

### Study selection

**Figure 1:**
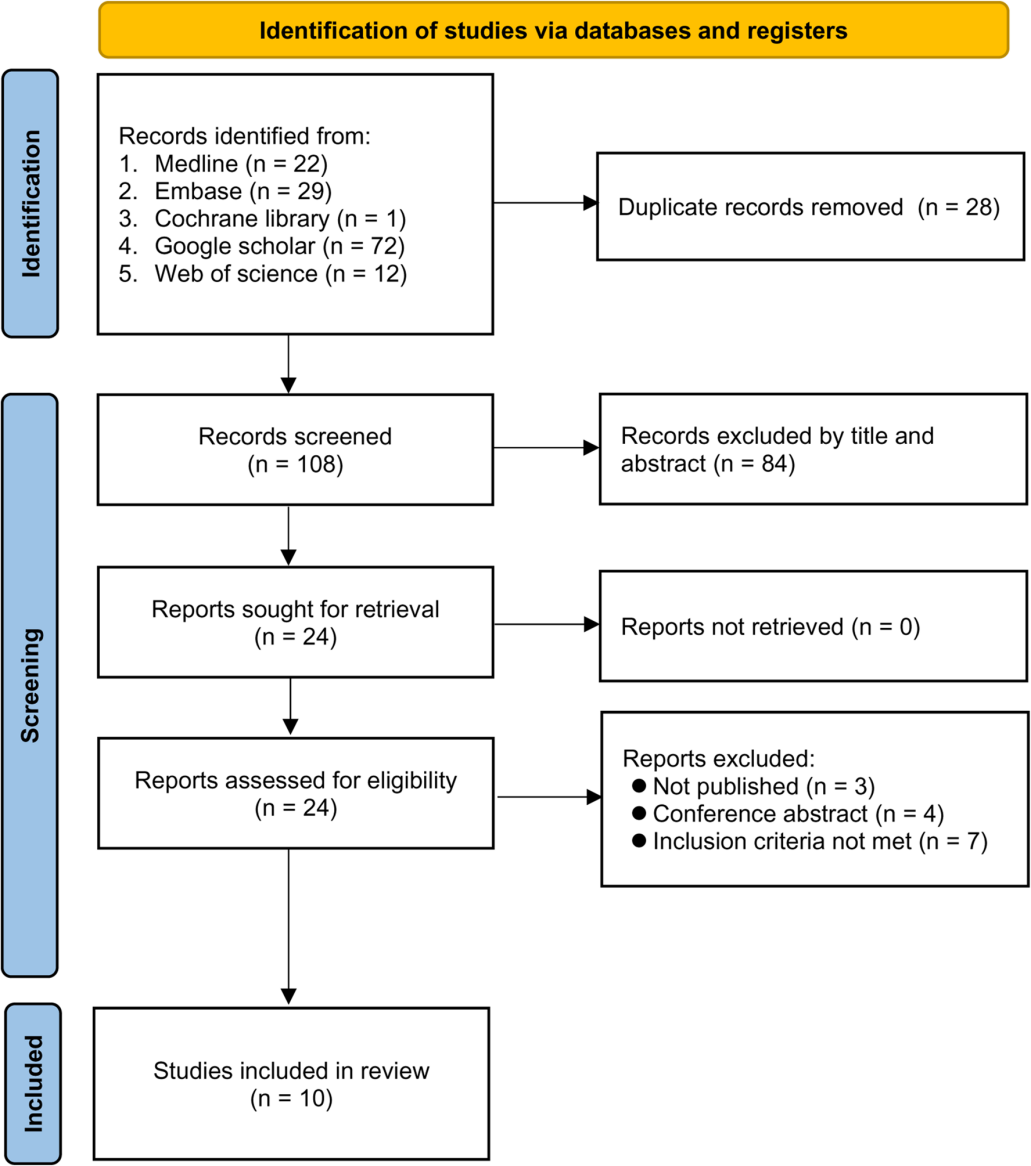
Summary of the process of study selection for the current meta-analysis.

A flow chart summarizing the process of study selection and exclusion is shown in Fig. 1. Of the 136 articles initially retrieved from the electronic databases, 112 were excluded due to duplicates (n = 28) or irrelevance (n = 84). Of the remaining 24 records assessed with a full-text review, 14 were excluded because of the informal nature of publication (i.e., Research Square) (n = 3), conference abstract (n = 4), and irrelevance (n = 7). Finally, a total of ten studies were included in the present meta-analysis^24–31,40,41^.

### Characteristics of included studies and risk of bias assessment

Ten studies involving 468 participants published between 2011 and 2022 were analyzed^24–31,40,41^. Characteristics of the studies including patient demographics, surgical setting, type of esophageal devices, and size of ETTs are described in Table 1. The number of patients in the included studies ranged from 13 to 80. Of the ten studies, seven reported on adult patients^24,26,27,30,40–42^, two focused on the pediatric population^25,28^, and one included both adult and pediatric patients^31^. The median or mean age of patients was between 0.5 and 66.8 years with a male prevalence of 28.3%-86.4%. One study did not offer detail regarding gender distribution^28^. Nine studies provided the BMI of the participants that ranged from 15.3 to 44.5 kg/m^2^, while this information was unavailable in the other study^25^. Seven studies involved cardiac surgery^24,26,28,31,40–42^, two investigated bariatric surgery^27,30^, and the other was on esophagogastroduodenoscopy^25^. One study, which examined the change in cuff pressure among patients undergoing endotracheal intubation with single and double lumen ETTs^29^, was split into two (i.e., Kim 2015a, Kim 2015b) to differentiate the study outcomes between the two devices. Focusing on the impact of age on cuff pressure, we divided the results of another report on changes in cuff pressure based on the age of the participants^31^ into five studies (i.e., Pan 2020a; Pan 2020b; Pan 2020c; Pan 2020d; Pan 2020e). Of the ten studies, nine observational studies compared the changes in cuff pressure before and after EDI^24–29,31,40,41^ and one was a randomized controlled trial^30^.

**Table 1:** Characteristics of included studies (n = 10).

Study (year)	Age (years)	Male (%)	BMI (kg/m^2^)	Procedure	ED	n	Airway device size	Definition of HCP (cmH_2_O)	Incidence of HCP	Mean CP change (cmH_2_O)	NOS
Tan 2011^24^	60.3	79	25.1	CS	TEE probe	38	7–7.5 mm¶	> 35	45%	8.5	8
Hung 2014^27^	34	28.3	37.7	BS	OG tube	60	7–8 mm¶	> 30	50%	8.3	8
Kim 2015^29^	66.8¶; 61.8‡	86.4¶ 59.1‡	23.4¶ 23.7‡	CS	TEE probe	44	7–7.5 mm¶, 32-39Fr‡	> 40	18.2%¶, 40.9%‡	6.9 12	8
Ozayar 2016^30^	38	35	44.5	BS	OG tube	40	7.5–8 mm¶	NA	NA	5.6	NA§
Balaban 2017^25^	11.3	50	NA	EGD	EGD probe	13	NA¶	NA	NA	5	8
Kamata 2017^28^	0.5–14	NA	15.3–20.3	CS	TEE probe	80	3–7 mm¶	> 30	22.50%	3.6	8
Borde 2020^26^	55	69.3	23.3	CS	TEE probe	65	7–8.5 mm¶	> 30	40%	8	8
Pan 2020^31^	2.9–24.6	60.3	22.2	CS	TEE probe	58	3–7.5 mm¶	> 30	23–77.5%	3–12.3	8
Maddali 2022^40^	55.7	73.5	28.7	CS	TEE probe	34	7.5–9 mm¶	NA	NA	20.3	8
Parajuli 2021^41^	40.5	61.1	23.6	CS	TEE probe	36	7–7.5 mm¶	> 30	50%	7.64	8

*BMI* body mass index; *EGD* esophagogastroduodenoscopy; *OG* orogastric tube; *TEE* transesophageal echocardiography; *HCP* high cuff pressure; *CP* cuff pressure; *BS* Bariatric surgery; *CS* Cardiac surgery; *NA* not available; *ED* Esophageal device; ¶single tracheal tube; ‡*DLT* double lumen tube; *NOS* Newcastle − Ottawa scale; §risk of bias assessed using the Cochrane Collaboration risk of bias tool.

Regarding the association of EDI with adverse effects, only airway complications were mentioned in the included studies. Eight studies reported no EDI-related complications (e.g., air leak or changes in ventilator parameters) after EDI^24–28,31,40,41^, while one study did not provide relevant information^29^. The other study reported an increased severity of sore throat in patients with EDI compared to those without at postoperative 30 min, 2 h, and 24 h^30^. No other ETT-associated respiratory complication or adverse events (e.g., esophageal injury) was reported in all studies.

The risks of bias of the nine observational studies are shown in Table 1. While the nine observational articles all showed a low risk of bias (i.e., total NOS score of 8 for each study), the risk of random sequence generation for the randomized controlled trial^30^ was deemed unclear because of a lack of specific information. In addition, the risk of bias for blinding of participants and personnel was high in this study^30^ as blinding of participants was impossible in this clinical setting.

### Study outcomes

#### Risk of high cuff pressure

**Figure 2:**
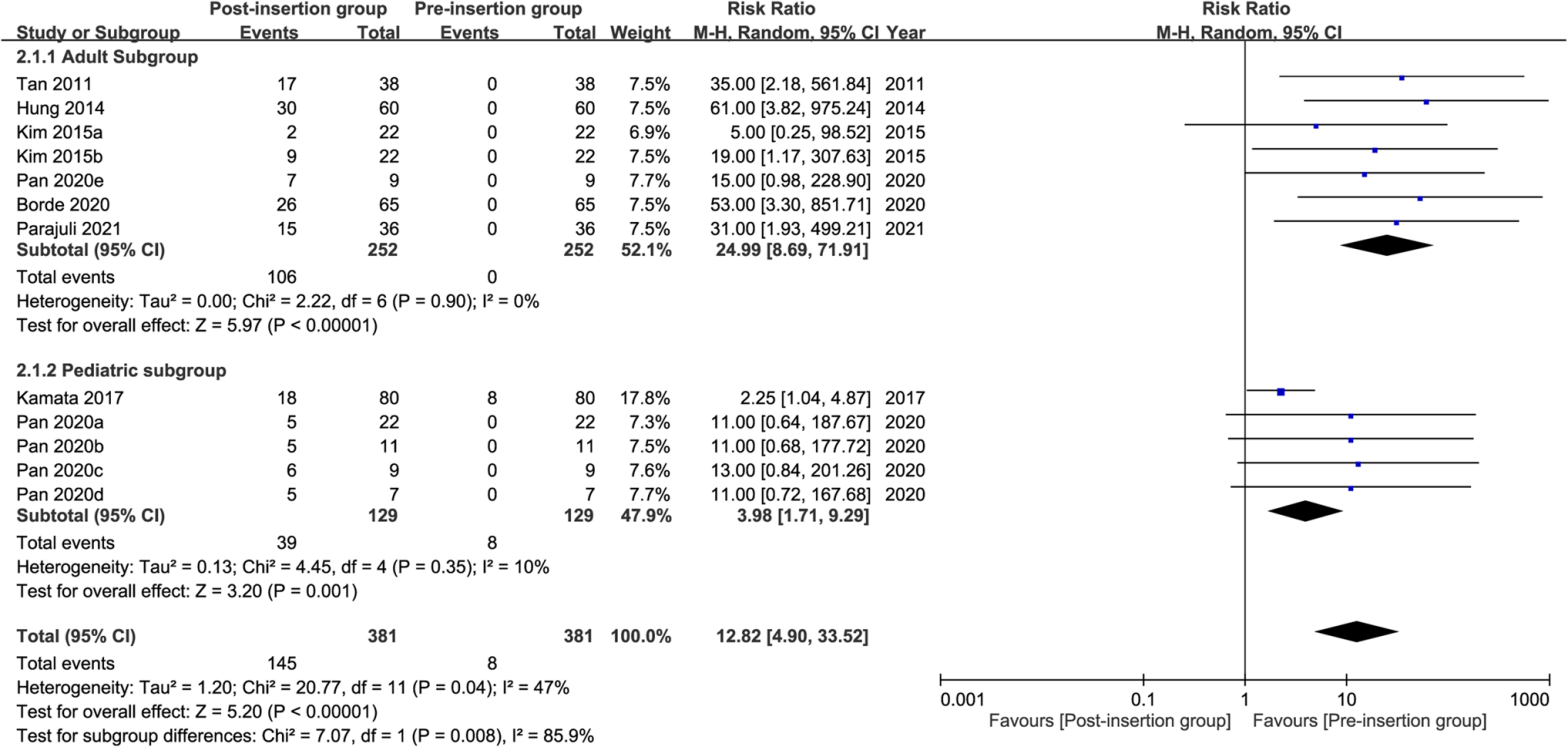
Forest plot comparing the risk of high cuff pressure after esophageal medical device insertion. *CI* confidence interval; *M–H* Mantel–Haenszel.

**Figure 3:**
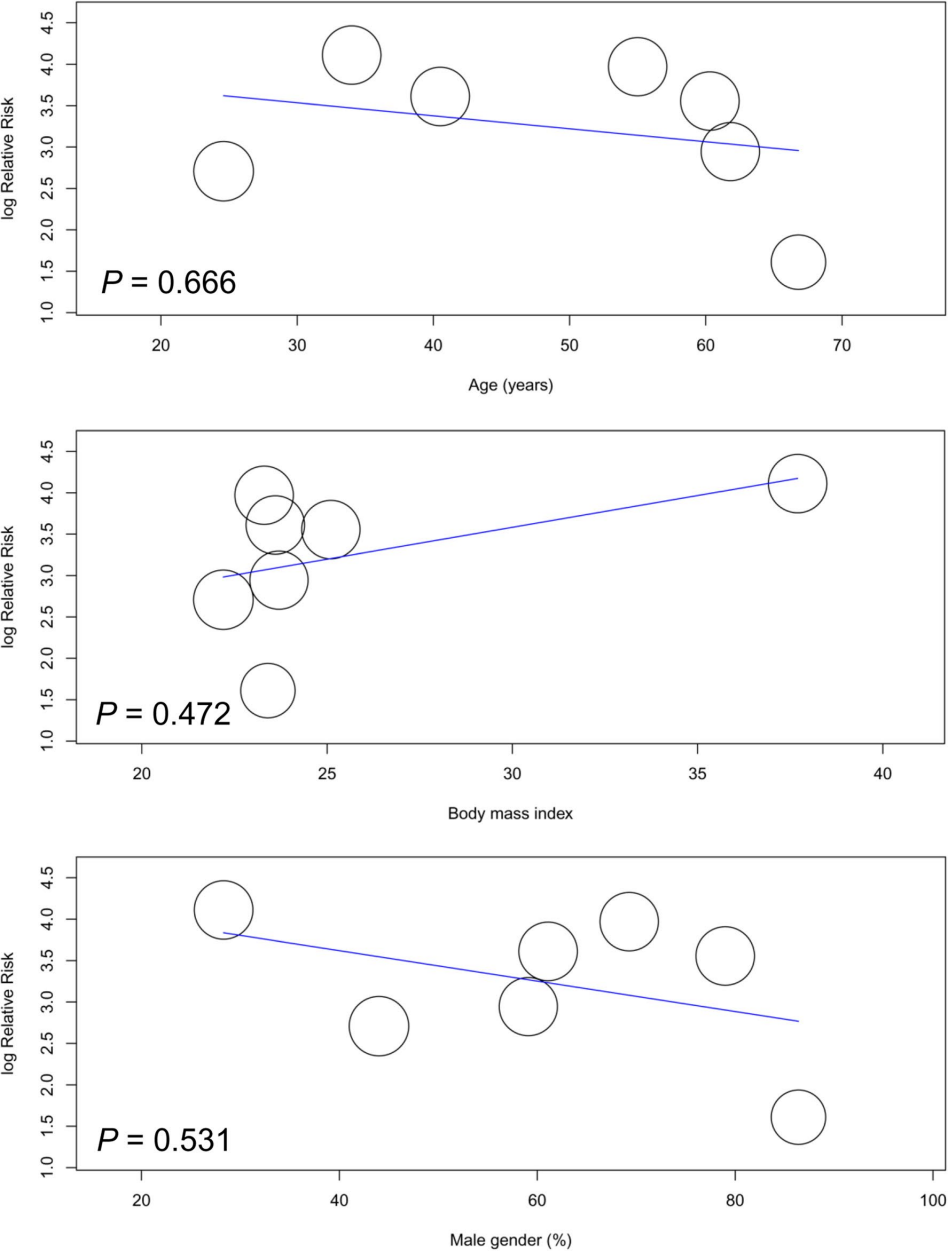
Meta-regression plot showing the association of patient characteristics (i.e., age, prevalence of male gender, and body mass index) with the risk of high cuff pressure.

**Figure 4:**
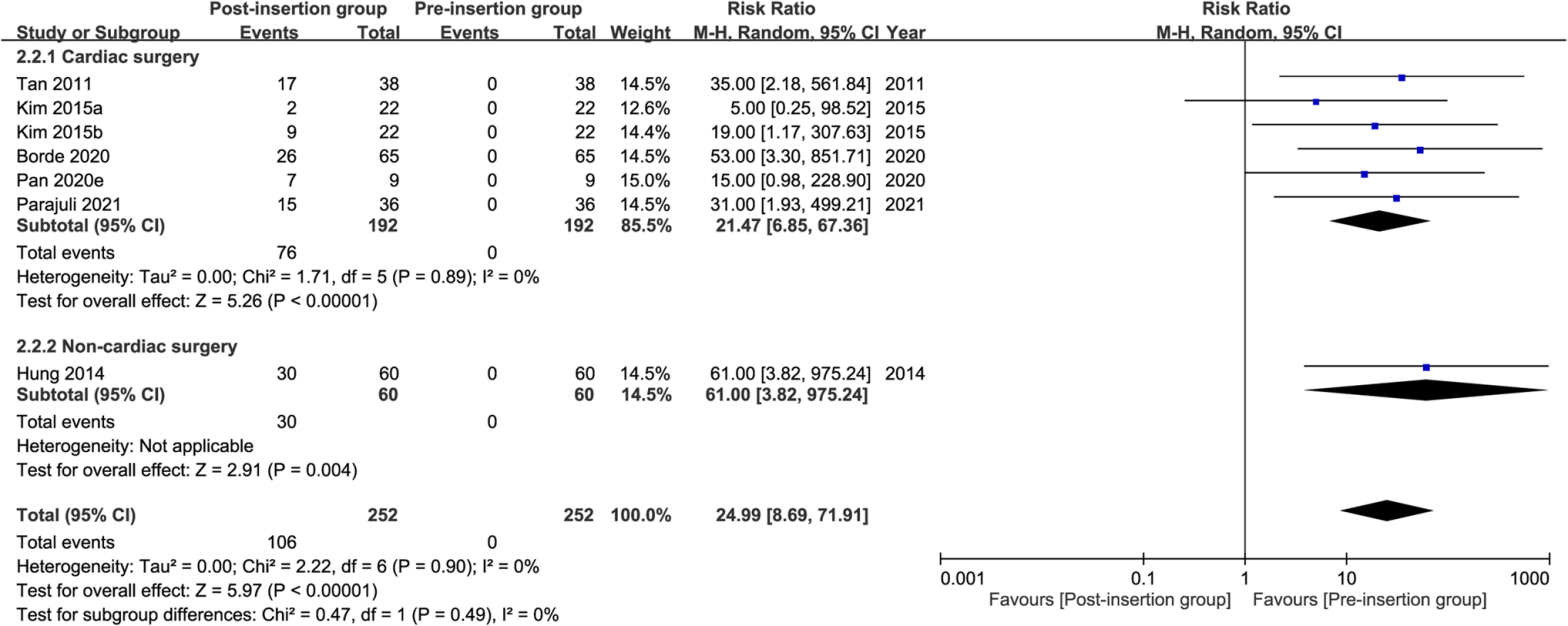
Forest plot comparing the risk of high cuff pressure after esophageal medical device insertion in adults undergoing cardiac and non-cardiac surgery. *CI* confidence interval; *M–H* Mantel–Haenszel.

Seven studies were available for the analysis^24,26–28,31,41,42^. A forest plot revealed a high risk of high cuff pressure following EDI (RR = 12.82, 95% CI 4.9 to 33.52, *p* < 0.00001; I^2^ = 47%) (Fig. 2). Subgroup analysis demonstrated a significant difference between adults and children (RR: 24.99 and 3.98 for adults and children, respectively, *p* = 0.008), implying a positive association between age and the risk of high cuff pressure. The heterogeneity within each subgroup among the included studies was low (i.e., I^2^ = 0% and 10% for adults and children, respectively). Sensitivity analysis showed no significant impact on outcome by omitting certain studies. For adult patients, meta-regression showed that age, prevalence of male gender, and BMI were not associated with the risk of high cuff pressure (Fig. 3). Similarly, the type of surgery (i.e., cardiac vs. non-cardiac) had no impact on the risk of high cuff pressure in adult patients (subgroup difference: *p* = 0.49)(Fig. 4).

#### Change in cuff pressure during esophageal device insertion

**Figure 5:**
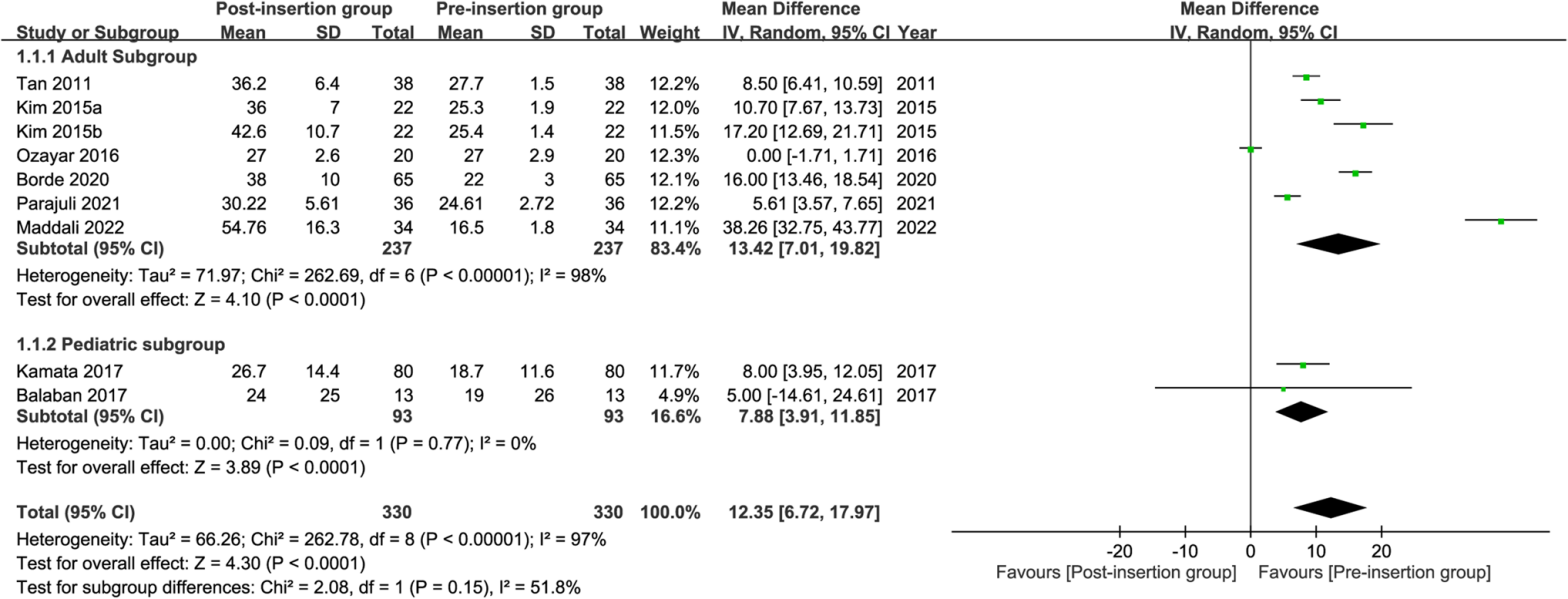
Forest plot for the comparison of changes in cuff pressure during esophageal medical device insertion. *CI* confidence interval; *IV* inverse variance.

Merged results from eight studies^24–26,28,30,40–42^ showed a significant rise in cuff pressure during EDI with increases in pressure being 13.42 and 7.88 cmH_2_O for adults and children, respectively (mean difference = 12.35 cmH_2_O, 95% CI: 6.72 to 17.97, *p* < 0.0001; I^2^ = 97%) (Fig. 5). Subgroup analysis found no significant difference between adults and children in this outcome (*p* = 0.15), suggesting that age was not significantly related to the change in cuff pressure during EDI. However, there was a high heterogeneity among the results from the adult subgroup across the included studies (I^2^ = 98%). Sensitivity analysis showed no significant impact on outcome by omitting certain studies.

#### Change in cuff pressure after esophageal device insertion

**Figure 6:**
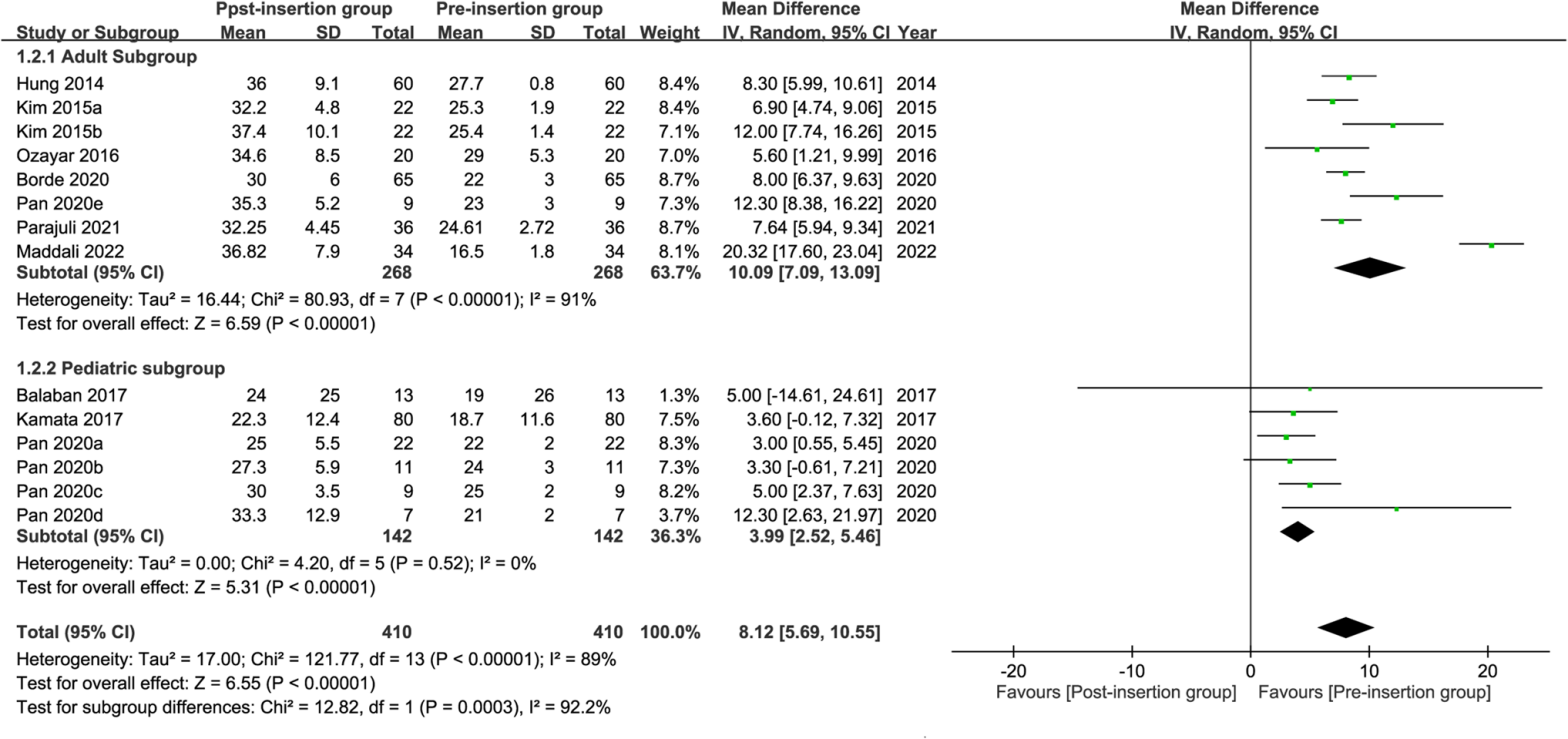
Forest plot for the comparison of changes in cuff pressure after esophageal medical device insertion. *CI* confidence interval; *IV* inverse variance.

The forest plot on the nine available studies^25–28,30,31,40–42^ demonstrated a significant elevation in tracheal cuff pressure following EDI in both adults and children (10.09 and 3.99 cmH_2_O, respectively) (pooled mean difference = 8.12 cmH_2_O, 95% CI: 5.69 to 10.55, *p* < 0.00001; I^2^ = 89%) (Fig. 6). Subgroup analysis revealed a significant difference between adults and children (*p* = 0.0003), indicating that age was a significant factor affecting the change in cuff pressure after EDI with an elevation in adults higher than that in children. The heterogeneity in the adult subgroup across the included studies was high (i.e., I^2^ = 91%). Sensitivity analysis showed no significant impact on outcome by omitting certain trials.

**Figure 7:**
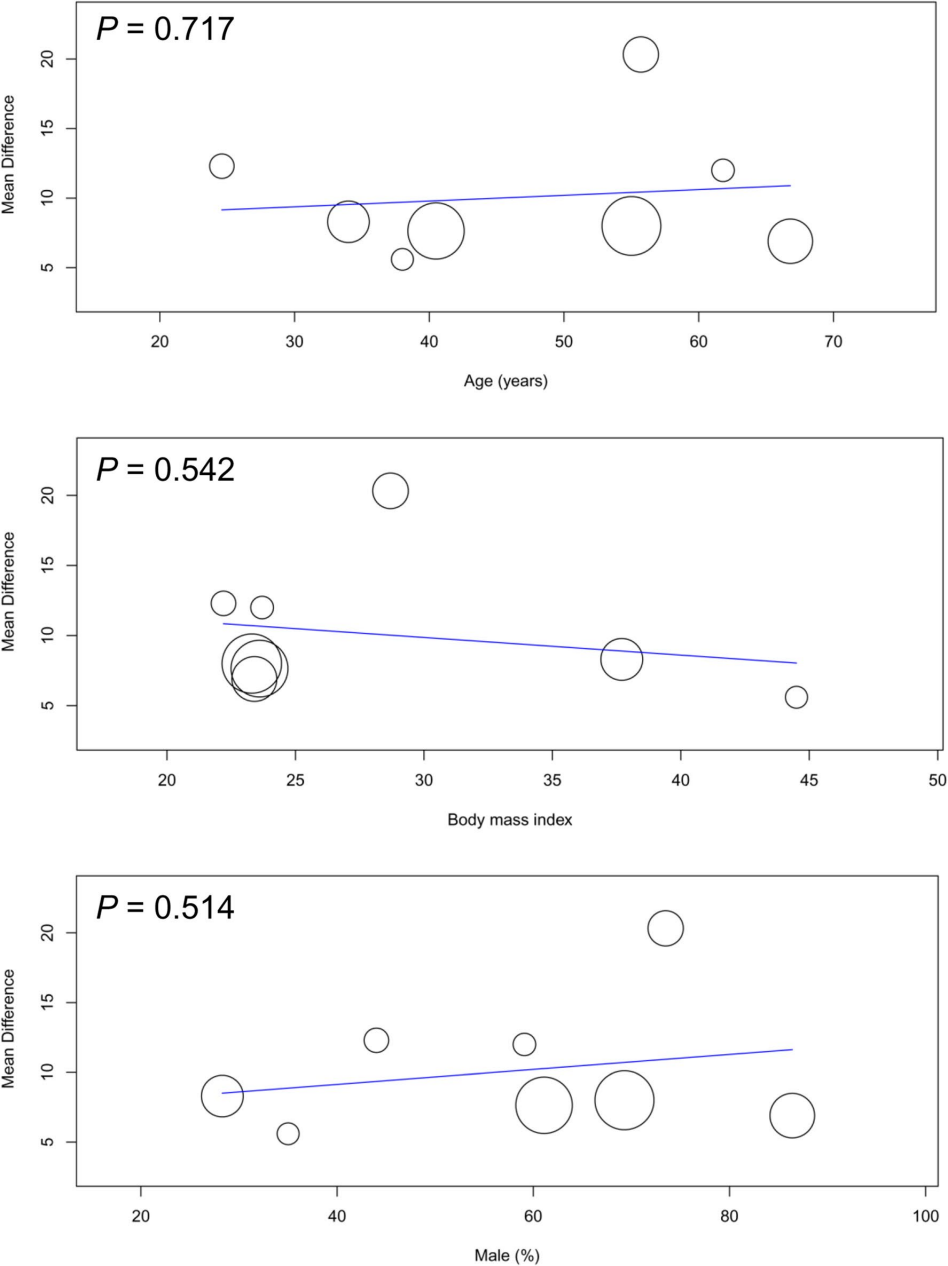
Meta-regression plot showing the association of patient characteristics (i.e., age, prevalence of male gender, and body mass index) with the mean difference in cuff pressure.

**Figure 8:**
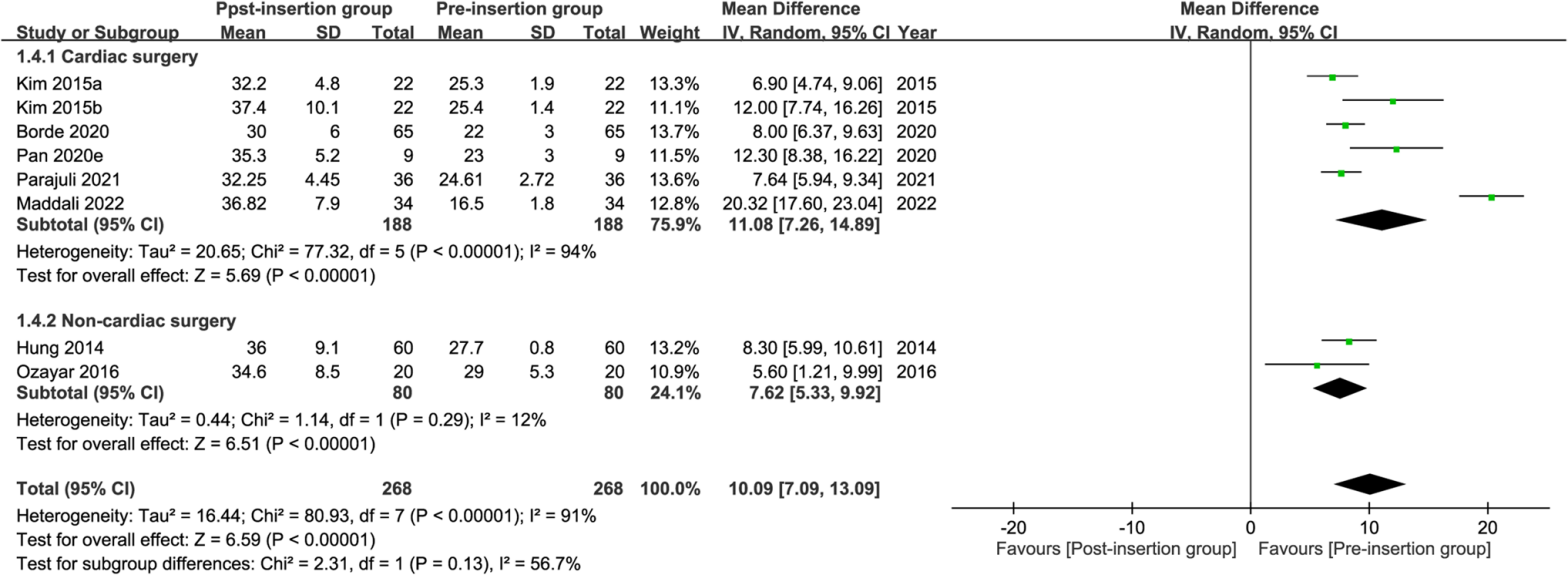
Forest plot for the comparison of changes in cuff pressure after esophageal medical device insertion in adults undergoing cardiac and non-cardiac surgery. *CI* confidence interval; *M–H* Mantel–Haenszel.

Meta-regression showed that the age, prevalence of male gender, and BMI were not correlated with changes in cuff pressure in adult patients (Fig. 7). Subgroup analysis in adult patients demonstrated that the type of surgery (i.e., cardiac vs. non-cardiac) had no impact on the changes in cuff pressure (subgroup difference: *p* = 0.13) (Fig. 8).

## Discussion

Despite the importance of maintaining the ETT cuff inflation pressure within an appropriate range^2–5^, the issue of changes in cuff pressure in procedures involving EDI was not systematically reviewed. To our best knowledge, the current meta-analysis is the first to address the impact of EDI on ETT cuff pressure based on available clinical evidence. Our results demonstrated an over 12-fold increase in risk of high cuff pressure (i.e., RR: 12.82) associated with the insertion of esophageal devices during endotracheal anesthesia. In addition, there were significant increases in cuff pressure both during and after device insertion in adults and children, highlighting the need for clinical concern regarding the impact of using esophageal devices on ETT cuff inflation pressure.

Previous studies have shown a prevalence of high cuff pressure in different clinical settings, including the operating theater in which the cuffs tend to be overinflated^43^, intensive care unit in which the cuff pressure may not be closely monitored^44^, and the emergency department in which physicians focus on life-threatening conditions^45^. The pressure inside the ETT cuff is also known to be affected by several factors, including lateral wall pressure, pneumoperitoneum^46^, duration of ETT placement^6^, patient position^47^, head position^48^, cuff position^49^, cuff volume, temperature^50^, use of nitrous oxide^51^, design of cuff^52^ and other less commonly reported factors.

Effort has also been made to modify the design of the cuff of ETT to minimize injury to the tracheal mucosa. To minimize the risk of overinflation associated with the conventional high-pressure low-volume cuff, the high-volume low-pressure ETT cuff has been introduced to avoid pressure-induced compromise of tracheal blood flow. However, previous studies showed that low-pressure cuffs may easily be overinflated to yield pressures that exceed capillary perfusion pressure^7^ for which cuff pressure monitoring is still vital.

On the other hand, the lack of accuracy of the two common methods of cuff pressure assessment, namely the fixed volume and pilot balloon palpation approaches^53,54^, may contribute to the high incidence of unnoticed cuff pressure elevation. A previous investigation has shown that actual measurement of the cuff pressure estimated by palpation with personal experience is often much higher than the optimal value when measured with manometry^52^. Although the minimal leak test has been introduced to evaluate cuff pressure, the accuracy of measurement is also questionable^55^. Another reason that may discourage the measurement of cuff pressure is the possible associated risk; it may lead to a drop in cuff pressure, which may cause leakage of secretions on the cuffs^56^.

The problem of cuff overinflation is further aggravated by the insertion of esophageal devices during endotracheal anesthesia when the cuff pressure is not usually monitored. Since the esophagus is in contact with the posterior membranous tracheal wall, introduction of an esophageal device (e.g., TEE probe) may directly compress the trachea and increase ETT cuff pressure, thereby compromising the microcirculation of trachea and its surrounding structures^24^. Indeed, a previous experimental investigation has demonstrated a notable negative impact of an increased cuff pressure on tracheal blood flow regardless of the duration^20^, underscoring the risk of adverse consequences from high cuff pressure triggered by esophageal device insertion for even a brief procedure. Therefore, manometry-guided control of cuff pressure has been proposed to reduce ETT-associated respiratory complications such as sore throat, hoarseness, cough, and blood-tinged expectoration even for procedures of short durations (i.e., 1–3 h)^52^. Nevertheless, although previous investigations have shown an increase in ETT cuff pressure induced by insertion of esophageal devices^24–31^, there was no pooled evidence identifying the risk of high cuff pressure and the net increases in pressure during and after device insertion as well as addressing the issue of difference between adults and children.

The choice of esophageal device may have a direct influence on cuff pressure. In the current study, there were three devices being introduced into the esophagus in the adult study population, namely the TEE probe, orogastric tube (i.e., bougie) for bariatric surgery, and the insertion tube of an endoscope. On the other hand, there was only one device used in children (i.e., TEE probe). Despite the relative noninvasiveness^57^ and usefulness of TEE probe as an intraoperative monitoring device for providing valuable information about the patient’s cardiac pathophysiological status^58^, it may be associated with the risks of pneumonia^59^ and other respiratory complications including airway compression and ETT malpositioning^57,60^. Moreover, cuff overinflation is another concern because monitoring of cuff pressure is not a routine practice among anesthetists during cardiac surgeries^61^. Consistently, an increase in cuff pressure may increase the risk of post-intubation tracheal stenosis in patients undergoing cardiac procedures^11^. Since TEE examination is usually performed in patients with hemodynamic instability, the hypotension–related reduction in tracheal mucosal perfusion pressure may further predispose patients to the risk of tracheal mucosal injury from tracheal cuff overinflation^29^. The use of orogastric tubes, which serve as both calibration tool and a volume reference device in bariatric surgery (e.g., laparoscopic sleeve gastrectomy), has also been implicated in respiratory tract complications such as arytenoid dislocation^62^ and hypopharyngeal perforation^63^. In addition to the reported elevation in the incidence of post-intubation sore throat in patients undergoing bariatric surgeries^64^ due to the likeliness of their need for difficult airway management^65^, the results of the current study suggest a possible further increase in the incidence of sore throat attributable to cuff overinflation if cuff pressure monitoring is unavailable.

In the current meta-analysis, we found a significantly higher risk of high cuff pressure in the adults (RR = 24.99) compared to the pediatric population (RR = 3.98). Nevertheless, a previous case report on a 12-year-old girl has demonstrated an association of EDI with airway obstruction^34^, highlighting the need for careful monitoring of cuff pressure as well as airway-related complications in the pediatric population. For adult patients, we recommended routine monitoring of cuff pressure because of our finding of an elevated risk of high cuff pressure after EDI.

A previous study that investigated the associations of the type of tracheal tube (i.e., single vs. double lumen), age, body height and weight as well as tracheal diameter with change in cuff pressure after EDI in adults using univariate analysis identified the type of tracheal tube as the only predictor^29^. In the current study, to assess the impact of demographic factors on change in cuff pressure in adult patients, univariate analysis with a meta-regression approach was conducted through including one covariate at a time (i.e., age, prevalence of male gender, BMI). The results showed that age, prevalence of the male gender, and BMI did not correlate with the risk of high cuff pressure and change in cuff pressure after EDI in adult patients. Therefore, our findings were consistent with those of that study^29^. In addition, our subgroup analysis on adults further demonstrated no significant impact of the type of surgery (i.e., cardiac vs. non-cardiac) on change in cuff pressure. These findings underscored the importance of routine monitoring of cuff pressure in adult patients regardless of their age, gender, BMI, and the type of surgery that they receive.

### Limitations

There were several limitations in the current study. First, the definitions of high cuff pressure varied among the included studies (e.g., > 30^26–28,31,41^, > 35^24^, or 40 cmH_2_O^42^. Therefore, the actual risk of high cuff pressure was underestimated in the present study when using > 30 cmH_2_O as a cut-off point for defining a high cuff pressure. Second, there was a high overall heterogeneity regarding the change in cuff pressure during EDI among the included studies probably attributable to the differences in patient population (i.e., adults vs. children) and the medical device chosen for each study. Previous studies have reported variations in cuff pressure with a number of factors including the size of ETT^66^, the design (e.g., shape) of the cuff^42,67^, and intubation time^68^ which, however, were not described in details in the included studies. Nevertheless, despite the possible variation in the size of ETT in children, the heterogeneity was acceptably low among the included studies. Finally, our results on children were only from three studies that used the same device (i.e., TEE probe) that may account for a relatively minor elevation in cuff pressure compared with that in adults so that the result could not be extrapolated to other devices in the pediatric population.

## Conclusions

The results of the current meta-analysis demonstrated an over 12-fold elevation in the risk of high endotracheal tube cuff inflation pressure especially in adult patients receiving esophageal device insertion under endotracheal anesthesia. There were significant increases in cuff pressure associated with the use of esophageal devices in both adults and children despite a higher increase in the former. The high heterogeneity across the included studies in the present investigation warrants further clinical trials to support its findings.

## Data Availability

The datasets used and/or analyzed in the current study are available from the corresponding author on reasonable request.
